# Transition Metal Catalyst Free Synthesis of Olefins from Organoboron Derivatives**

**DOI:** 10.1002/chem.202104125

**Published:** 2022-02-28

**Authors:** K. Bojaryn, C. Hirschhäuser

**Affiliations:** ^1^ Institut für Organische Chemie Universität Duisburg-Essen Universitätsstraße 7 45141 Essen Germany

**Keywords:** boronic ester, boron Wittig, carbenoid, highly substituted alkene, olefination

## Abstract

Stereoselective preparation of highly substituted olefins is still a severe challenge that requires well defined elimination precursors. Organoboron chemistry is particularly suited for the preparation of molecules with adjacent stereocenters. As organo boron substrates with leaving groups in β‐position can undergo stereospecific *syn*‐ or *anti*‐elimination, this chemistry harbors great potential for the synthesis of complex olefins. In recent years three main strategies emerged, which differ in their approach to the β‐functionalized organoboron elimination precursor. (i) Stereoselective preparation of such elimination precursor can be achieved by addition of a boron‐stabilized anion (d^1^) to an aldehyde or ketone (a^1^) or diastereoselective 1,3‐rearrangement of suitable boron‐ate‐complexes. Stereospecific methods rely either on (ii) diastereospecific 1,2‐metalate rearrangement of boron‐ate‐complexes that involve opening of appropriate heterocycles or (iii) addition of chiral carbenoids (d^1^*) to chiral boronates (a^1^*) with a leaving group in α‐position.

## Graphical abstract

**Many boron based olefinations** proceed via intermediates with leaving groups in the β‐position. These can undergo stereospecific *syn*‐ or *anti*‐elimination and are useful for the synthesis of complex olefins. Three main strategies emerged differing in the preparation of the pivotal intermediate: (i) Diastereospecific methods like the addition of a boron‐stabilized anion (d^1^) to an aldehyde or ketone (a^1^), (ii) Diastereospecific 1,2‐metalate rearrangements of boron‐ate‐complexes with appropriate heterocycles and (iii) addition of chiral carbenoids (d^1^) to boronates with a suitable group in α‐position (a^1^).

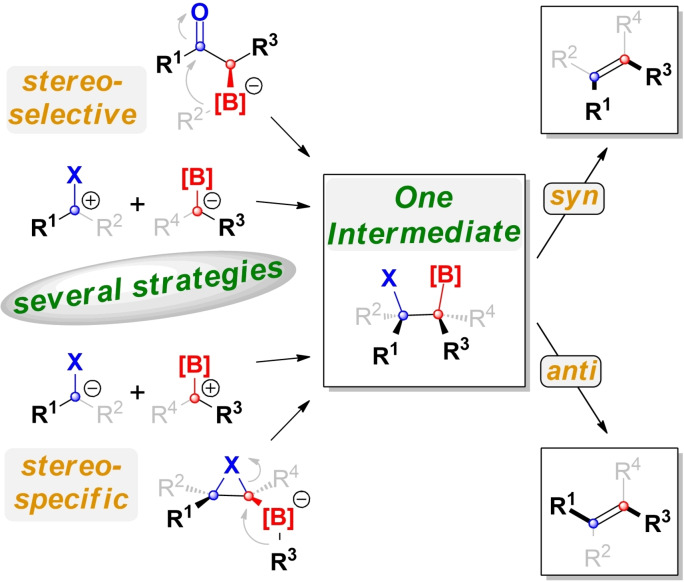

## 1 Introduction

**Scheme 1:**
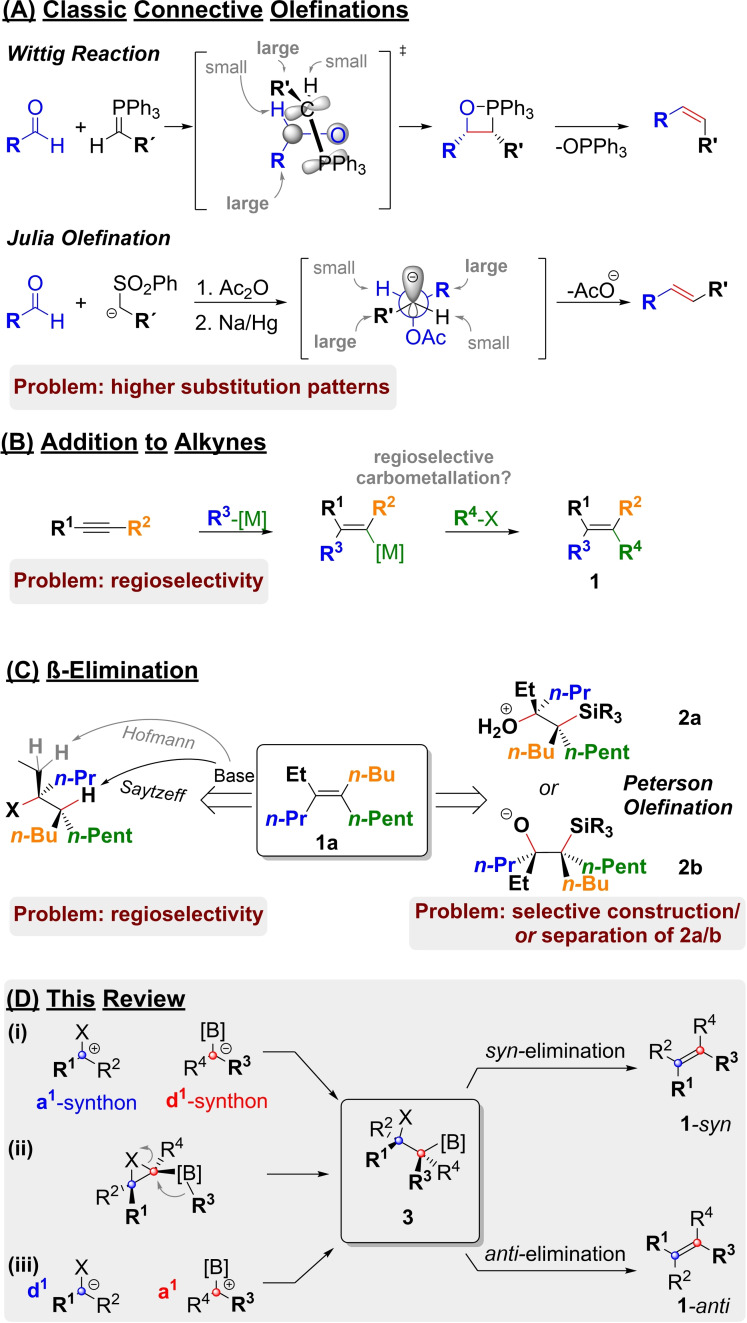
Textbook routes to olefins have problems with higher substitution patterns (**1**) or the selective synthesis of silyl elimination precursors (**2**). Corresponding boronates (**3**) have become readily available and can serve as suitable substitutes.

The stereoselective synthesis of highly substituted olefins is still a challenging task in organic chemistry.^[1]^ To illustrate the underlying problems some classical strategies for olefin syntheses and their associated problems are depicted in Scheme 1. Disubstituted alkenes can be prepared with good *E*‐/*Z*‐selectivity by classical methods such as the Wittig‐^[2]^ or Julia‐olefination^[3]^ (Scheme 1A). However, in higher substituted olefins sterical hindrance in the transition state, as well as the final product, hampers alkene formation.^[6]^ This low reactivity can be overcome with more reactive methods (e. g. HWE^[4]^ or McMurrey^[5]^). However, all carbonyl based connective olefinations encounter a fundamental problem of stereoselectivity when the synthesis of higher substitution patterns is attempted. At the end of the day, the selectivity of these reactions depends on the sterical and/or electronical differences of the aldehyde's and/or the ylide's/sulfone's substituents.^[6]^ Thus, higher substitution patterns with similar substituents cannot be prepared stereoselectively with these methods. A common alternative is the utilization of organometallic chemistry for olefin synthesis. Carbometalation of alkynes, followed by alkylation or cross coupling of the addition products can lead to highly substituted alkenes (**1**, Scheme 1B).^[7]^ While the carbometalation step is indeed stereospecific, its regioselectivity depends on the nature of the substituents R^1^ and R^2^. As carbometalation of alkenes suffers from the same issue, similar limitations are met in Heck couplings as well.^[8],[9],[10]^ In both cases good results in the synthesis of highly substituted alkenes were achieved, with anion stabilizing groups at R^2^ steering carbometalation. However, the stereodefined synthesis of highly substituted alkenes with sterically and electronically similar substituents like **1 a** is not possible by either of these methods. Stereospecific elimination of well‐defined precursors as shown in Scheme 1C is a potential solution for the problem. However, simple elimination of H−X suffers from classic problems of regioselectivity (Hofmann vs. Sayzeff).^[1]^ The Peterson olefination^[11]^ avoids this issue by strictly defining the elimination partners (i. e. the hydroxyl‐ and the silyl group), as well as the elimination mechanism (*syn* vs. *anti*). Unfortunately, this requires either the stereoselective synthesis of the corresponding precursors of type **2**, or at least their separation. Fortunately, β‐elimination of boranes and boronic esters (R_n_B−X) can be achieved in a manner similar to the elimination of R_3_Si−X (Scheme 1D).^[12]^

As boronic esters have been used extensively (e. g. in conjunction with carbenoids) for the stereoselective synthesis of adjacent stereocenters (**3**), a great potential for the synthesis of stereochemically well defined olefins arises.^[13]^ Suitable elimination precursors of type **3** can be synthesized in a stereoselective manner, before they are subjected to either stereospecific *syn*‐, or *anti*‐elimination.

For the purpose of this review, we will categorize these methods based on the synthesis of the pivotal elimination precursor **3**. Therefore, three approaches are distinguished: (i) stereoselective methods, such as the addition of boron‐stabilized anions to suitable a^1^‐building blocks^[14]^ (i. e. aldehydes), (ii) stereospecific 1,2‐metalate rearrangement of boron‐ate‐complexes under opening of three membered heterocycles and (iii) a stereospecific umpolungs‐strategy, that employs chiral carbanions and boronates with a leaving group in α‐postion (i. e. a chiral a^1^‐synthon).

In the following sections we discuss key examples for these approaches, as well as selected results from other publications on organoboron chemistry, that might help to overcome some of the current limitations.

## 2 Diastereoselective Olefinations

### 2.1 Diastereoselectivity Based on Addition to a^1^‐Reagents

**Scheme 2:**
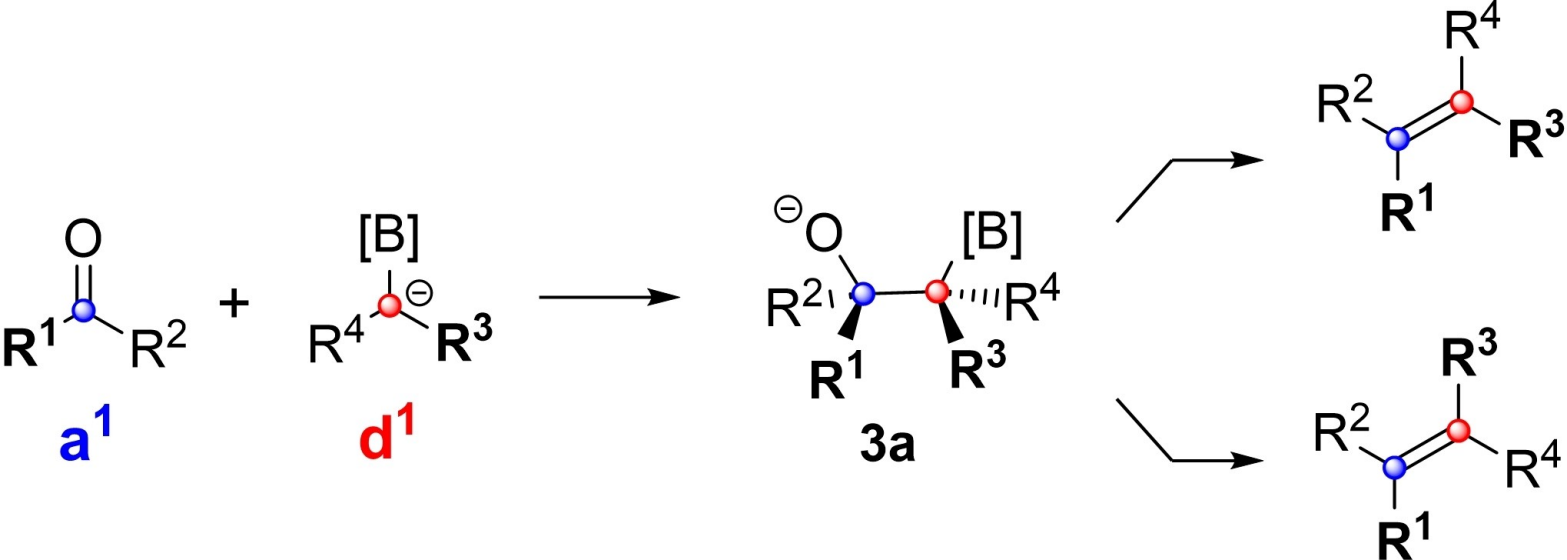
Addition of boron‐stabilized anions to carbonyl groups.

This first section starts with a discussion of the addition of boron‐stabilized anions (i. e. d^1^‐reagents) to aldehydes and ketones (i. e. a^1^‐building blocks). When followed by elimination, this reaction is referred to as the boron‐Wittig reaction (Scheme 2).^[15]^ The diastereoselectivity of the initial addition is often critical for the overall *E/Z*‐selectivity. Therefore, a short discussion of models that help to predict said selectivity is in order.

**Figure 1:**
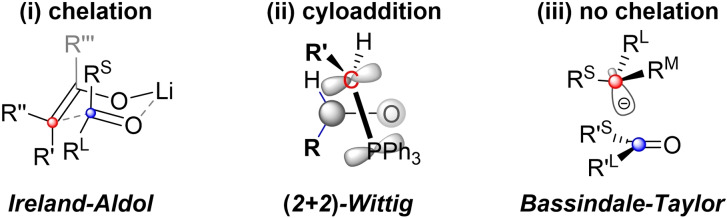
Models for predicting the diastereoselective addition of chiral carbanions (red) to carbonyl groups (blue).

In principle three different types of transition state models for the addition of prochiral carbanions to prochiral carbonyl groups can be distinguished as shown in Figure 1: (i) chelation‐based models, such as the classic Zimmerman‐Traxler‐model^[16]^ usually look at the cyclic transition states, from which the products arise. However, these models are usually not invoked for boron‐Wittig reactions. (ii) After long disputes a (2+2)‐cycloaddition mechanism has been widely accepted for Wittig reactions with destabilized ylides.^[6]^ In principle a similar transition state could play a role in some boron‐Wittig reactions as well. However, as will be discussed in the following section, there is good evidence for the existence of open structures of type **3**. While this does not exclude a (2+2)‐cycloaddition mechanism, it would at least brand the resulting bora‐oxetane as an unstable intermediate. (iii) A simple empirical model for the addition of prochiral anions to prochiral aldehydes has been suggested by Bassindale and Taylor.^[17]^ As shown in Figure 1, the model postulates that the anion will approach the carbonyl group in a way that places the smallest substituent (R^S^) between the two carbonyl‐substituents. Furthermore, the largest substituent (R^L^) of the carbanion is positioned on the same side as the smallest substituent of the carbonyl derivative (R’^S^). This automatically places the medium sized carbanion substituent (R^M^) next to the largest substituent of the carbonyl compound (R’^L^). It should be noted that this simple empirical model also correctly predicts the outcome of the Wittig‐reaction with electron rich ylides. But although it might sometimes give the right result for the wrong reason, the Bassindale‐Taylor model nevertheless is a great aid for understanding boron‐Wittig reactions.

**Scheme 3:**
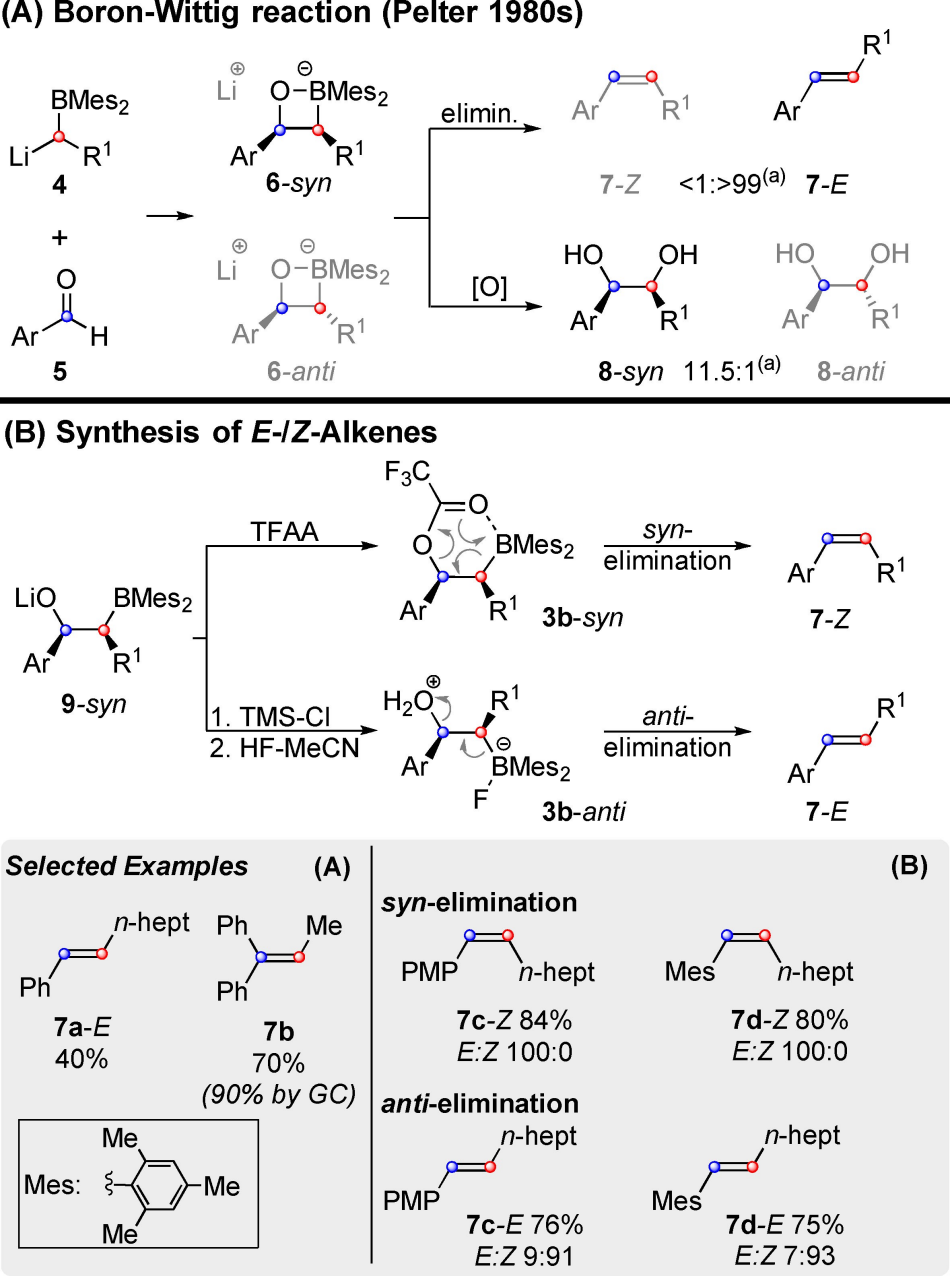
Synthesis of diols and alkenes from lithiated dimesitylboranes and aldehydes/ketones: the boron‐Wittig reaction. (a) exemplary diastereoselectivity for Ar=Ph and R^1^=heptyl. Mes=Mesityl, PMP=*para*‐Methoxyphenyl.

First varieties of the well‐known Wittig reaction^[18]^ using boron^[19]^ or sulphur^[20]^ instead of phosphorus, were reported in the 1960s. In the 1980s Pelter and coworkers explored the “boron‐Wittig” reaction and showed its mechanistic potential in analogy to the “silyl‐Wittig” reaction (i. e. the Peterson olefination).^[21]^ As shown in Scheme 3A, reaction of lithiated dimesitylboranes (**4**) with aromatic aldehydes (**5**) led to alkenes of type **7**. Depending on the substrates, this method is usable even at room temperature and preferably delivers *E*‐alkenes such as **7 a**‐*E*. Furthermore, reaction with non‐enolisable and symmetrical ketones provided trisubstituted alkenes such as **7 b** in good yield. Reactions with enolisable ketones and aldehydes on the other hand proceed poorly at higher temperatures, but delivered up to 80 % yield at ‐78 °C. Initially the preference for *E*‐alkenes of type **7**‐*E* led the researchers to the conclusion that the product must arise from oxaboretane **6**‐*anti*, by *syn*‐elimination, just as in a classic Wittig‐reaction. However, in later studies^[21b]^ they showed that alkaline oxidation of the addition product **6** primarily produced diol **8**‐*syn*. As the oxidation reaction proceeds under retention of configuration,^[21]^ **8**‐*syn* must have been formed from **6**‐*syn*. This means that alkene **7**‐*E* must have been formed by *anti*‐elimination. The preferred formation of oxaboretanes **6**‐*syn*, thus mirrors the Wittig reaction (with electron rich substrates), in which *syn*‐oxaphosphetanes are formed more swiftly. Due to the shorter length of the C−B‐bond, bora‐oxetanes of type **6**, might well be better represented by the open structure **9**, which can rotate freely and thus undergo different types of elimination. This was exploited by Pelter as shown in Scheme 3B.^[21c]^ On the one hand the preferred addition product **9**‐*syn* was trapped with trifluoroacetic anhydride (TFAA). S*yn*‐elimination via a six membered transition state generated *Z*‐alkenes (**7 c/d** *‐Z*). On the other hand, intermediates of type **9** *‐syn* could also be trapped with TMS−Cl at −78 °C. Subsequent *anti*‐elimination was triggered by the addition of aqueous HF‐MeCN, thus delivering the corresponding *E*‐alkenes (**7 c/d** *‐E*). Later investigations^[21]^ showed, that only benzylic aldehydes can undergo *syn*‐elimination, when TFAA is used as a trapping agent. Aliphatic aldehydes can be turned into alkenes in the presence of protic acids (HX).^[21e]^ However, their elimination cannot be steered along the lines of the two stereospecific pathways that easily. Very sterically hindered aldehydes predominately formed *Z*‐olefins, although the use of strong acids (e. g. HCl) increased the amount of *E*‐alkenes distinctively. Although Pelter's work on the boron‐Wittig reaction did not lead to widespread adoption due to its limited substrate range, it nevertheless serves as an excellent example of the key concept (Scheme 1): a stereoselective synthesis of precursors of type **3**, followed by a stereospecific elimination. A major discovery of Pelter was that oxaboretanes (**6**) are better described as β‐alkoxy boranes (**9**), which can freely rotate around the newly formed C−C‐bond. This fact is particularly relevant when bis(boryl)methane derivatives are employed, as discussed in the following section.

### 2.2 Selectivity based on Diastereoselective Elimination

**Scheme 4:**
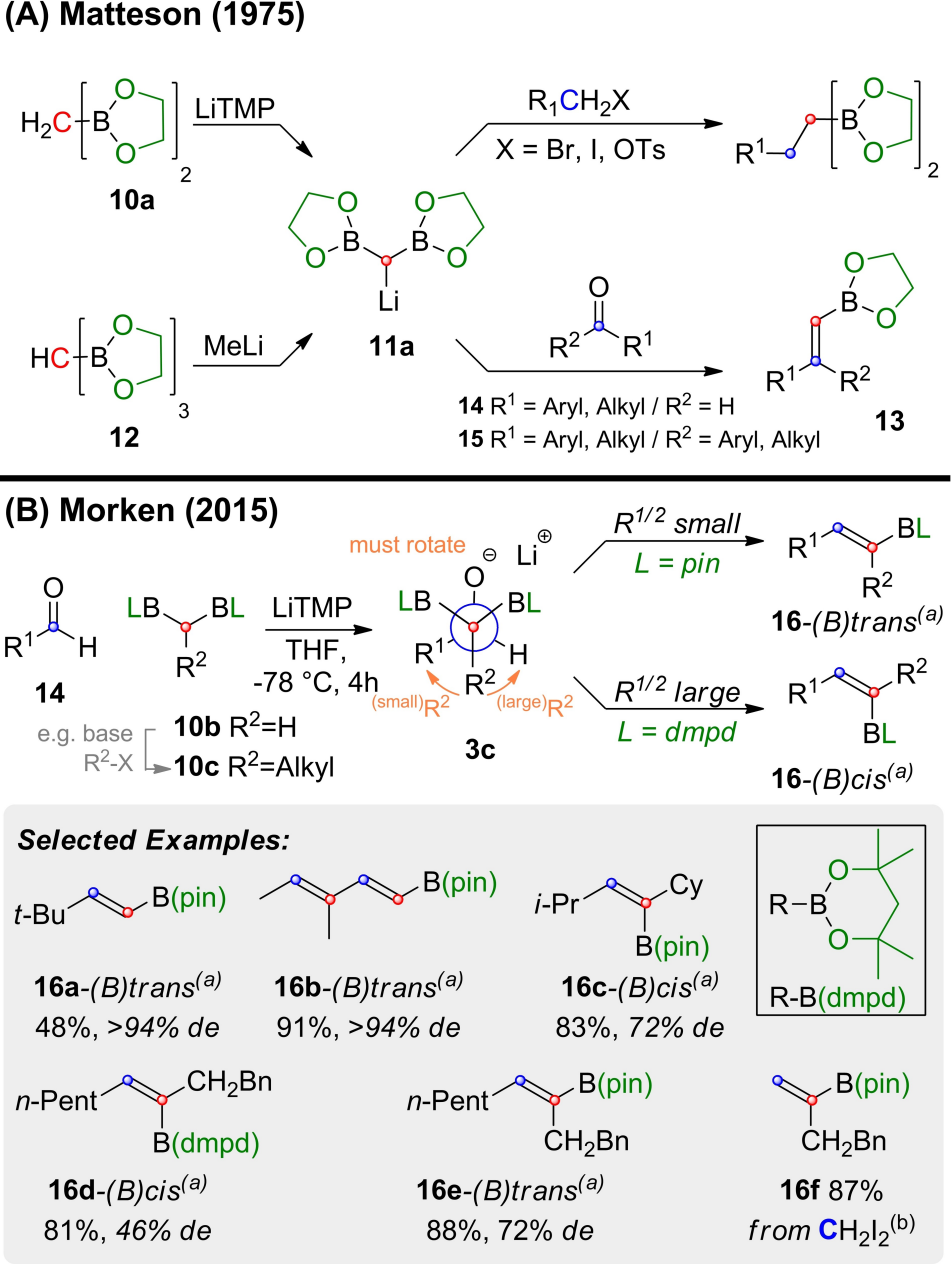
Synthesis of vinyl boronic esters by boron‐Wittig reaction. (a) The descriptors *(B)trans* and *(B)cis* are used in a similar manner as in enolate‐nomenclature, i. e. *(B)trans* and *(B)cis* always refers to the relative position of the boronic ester and the vincinal substituent with the highest priority (R^1^). (b) Conditions for the preparation of **16 f** deviated: LiTMP, THF, 0 °C, 5 min, then CH_2_I_2_ (2.0 equiv.), 0 °C to 60 °C 2 h. dmpd=dimethylpentadiethylato.

As shown in Scheme 4A bis(boryl)methane derivatives of type **10** can be lithiated readily with sterically hindered LiTMP and then be employed for boron‐Wittig chemistry. Matteson and coworkers^[22]^ generated the lithiated species **11 a** either by deprotonation of glycolester **10 a**,^[22d]^ or anionic deborylation of tris(ethylenedioxyboryl)methane **12**.^[22a]^ The group successfully explored both alkylation of **10 a** ^[22d]^ and its boron‐Wittig reaction with aldehydes (**14**) and ketones (**15**) yielding vinyl‐boronates of type **13**. The group also found that a complexing agent for lithium cations such as DABCO (diazabicyclooctane) and HMPA (hexamethylphosporamide) or TMEDA (tetramethyl‐ethylenediamine) aids reactivity.^[22d]^

Unfortunately, the glycolester **10 a** is hydrolytically sensitive and its preparation not trivial. In 2015 Morken and coworkers adapted the reaction to the commercially available pinacol ester **10 b** ^[23]^ from which the synthesis of alkyl substituted derivatives of type **10 c** is straightforward (Scheme 4B).^[22]^ By reacting these substrates with suitable aldehydes and LiTMP in THF substituted vinyl‐boronic esters (**16**) were readily available with impressive stereoselectivity. When the substituents on the aldehyde (R^1^) and the *bis*‐boronate (R^2^) were small, the (B)‐*trans* isomers (**16**‐*(B)trans*) were formed predominately (**16 a‐b**). However, when both R^1^ and R^2^ were large, the selectivity was inverted and isomers of type **16**‐*(B)cis* predominated (**16 c**‐*(B)cis*). The latter trend could be intensified by using dimethylpentanediolato (dmpd) boronic esters. In some cases, this even allowed for an inversion of stereoselectivity, as can be seen in **16 d**‐*(B)cis* and **16 e**‐*(B)trans*, in which the exchange of the boronic ester led to an inversion of diastereoselectivity. Products that would formally require the use of formaldehyde like **16 f**, were prepared from CH_2_I_2_.

The stereoselectivity observed for **16 a‐e** can be rationalized with the help of the Bassindale‐Taylor model. It predicts that **3 c** is formed in the conformation depicted in Scheme 4. As both boronic esters are in principle available for elimination the stereoselectivity of the reaction must arise in the elimination step (**3 c→16**). *Syn*‐elimination of one of the two diastereotopic boronic esters (BL) requires rotation of **3 c** around the newly formed C−C bond. That also means that either R^2^, or one of the boronic esters (BL) must rotate into a *syn*‐periplanar conformation relative to R^1^. This explains why the relative bulk of these two substituents is a key factor for the stereoselectivity observed in the formation of **16 a**–**e**.

**Scheme 5:**
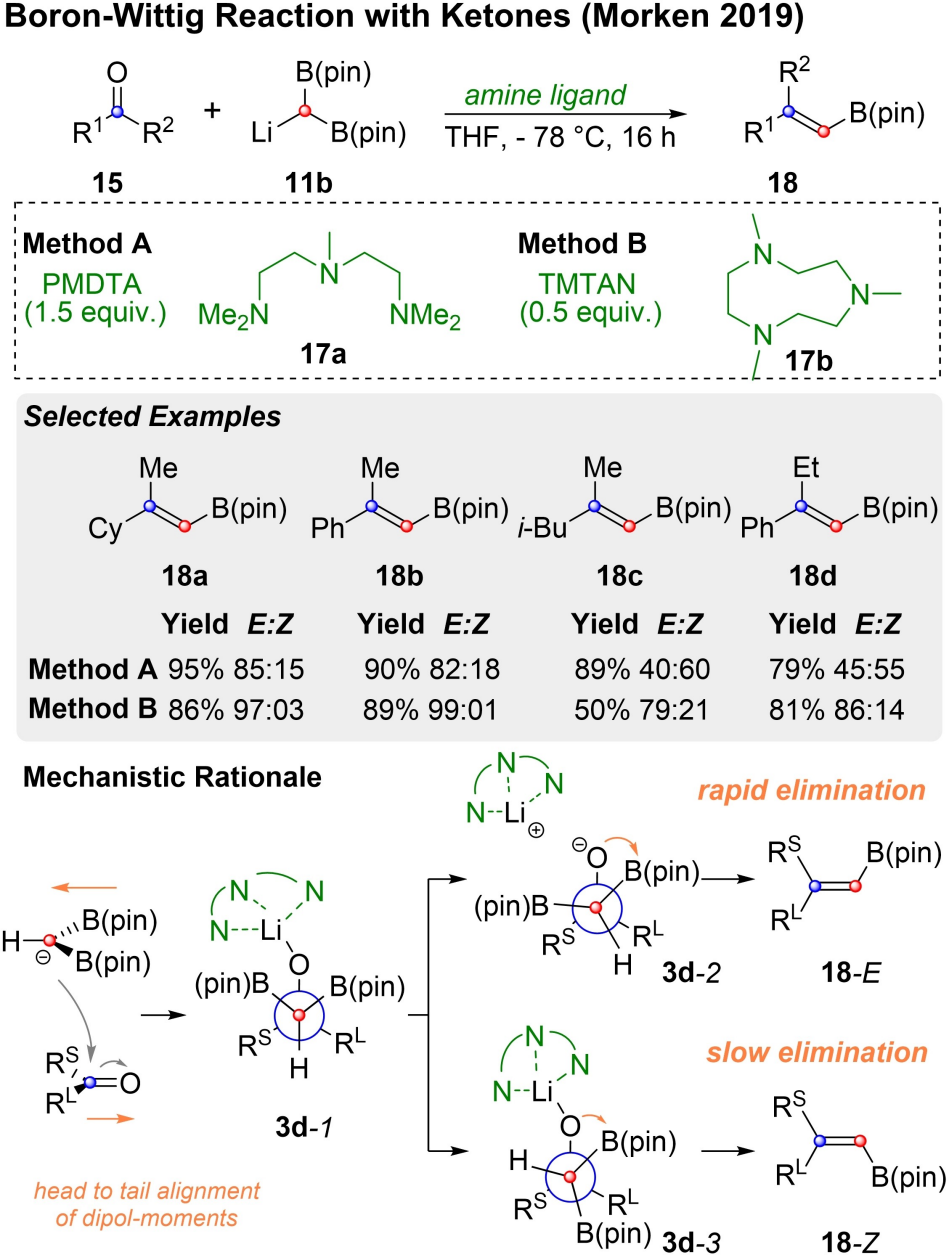
The stereoselective synthesis of vinyl‐boronic esters by boron‐Wittig reaction with ketones was successfully realized by Morken and coworkers using diamine ligands.^[23]^ The group also suggested a mechanistic rational which extends the Bassindale‐Taylor model.

In 2019 the Morken group extended this concept to the stereoselective boron‐Wittig reaction of lithiated bis(pinacolatoboryl)methane (**11 b**) with ketones (**15**) that carry substituents with a distinguished steric bias (Scheme 5).^[23]^ As already recognized by Matteson,^[22]^ the presence of amine ligands can be beneficial in boron‐Wittig reactions. To a certain extent, in situ preparation of **11 b** with LiTMP already provides a decent amino ligand (TMP^−^), but Morken and coworkers tested several other tri‐ and tetradentate amines, in order to optimize the *E/Z* ratio of this challenging reaction. The best results were obtained when **11 b** was prepared in a ligand free manner and PMDTA (1.5 equiv., method A) or TMTAN (0.5 equiv., method B) were added subsequently. Products like **18 a** and **18 b** were mainly obtained as *E*‐alkenes, independently of the method. For other substrates the choice of the amine additive was much more important and could even invert diastereoselectivity (**18 c** and **18 d**). In order to explain this intriguing observation, Morken and co‐workers invoked the Bassindale‐Taylor model, which initially predicts the formation of **3 d** *‐1*. According to Reich and coworkers both PMDTA (**17 a**) and TMTAN (**17 b**) divide dimeric Li‐enolates into monomeric moieties, with the latter being the more efficient ligand.^[24]^ Greater dissociation of the O−Li bond in the addition product leads to faster *syn‐*elimination, via conformation **3 d**‐*2*, which is formed directly from **3 d**‐*1* by rotating one of the boronates towards the smaller substituent R^S^. With a less efficient ligand, like PMDTA, dissociation of the O−Li bond takes place to a lesser extent, which slows down *syn*‐elimination and stabilizes the addition products **3 d**. Therefore, complete bond rotation has enough time to occur. From this Curtin‐Hammet situation Morken suggests that elimination preferably occurs from conformation **3 d** *‐3*. Here, the bulky amine ligand is positioned on the side of the smaller substituent (R^S^), which results in the positioning of the remaining B(pin) group on the other side, i. e. next to the larger substituent R^L^.

**Scheme 6:**
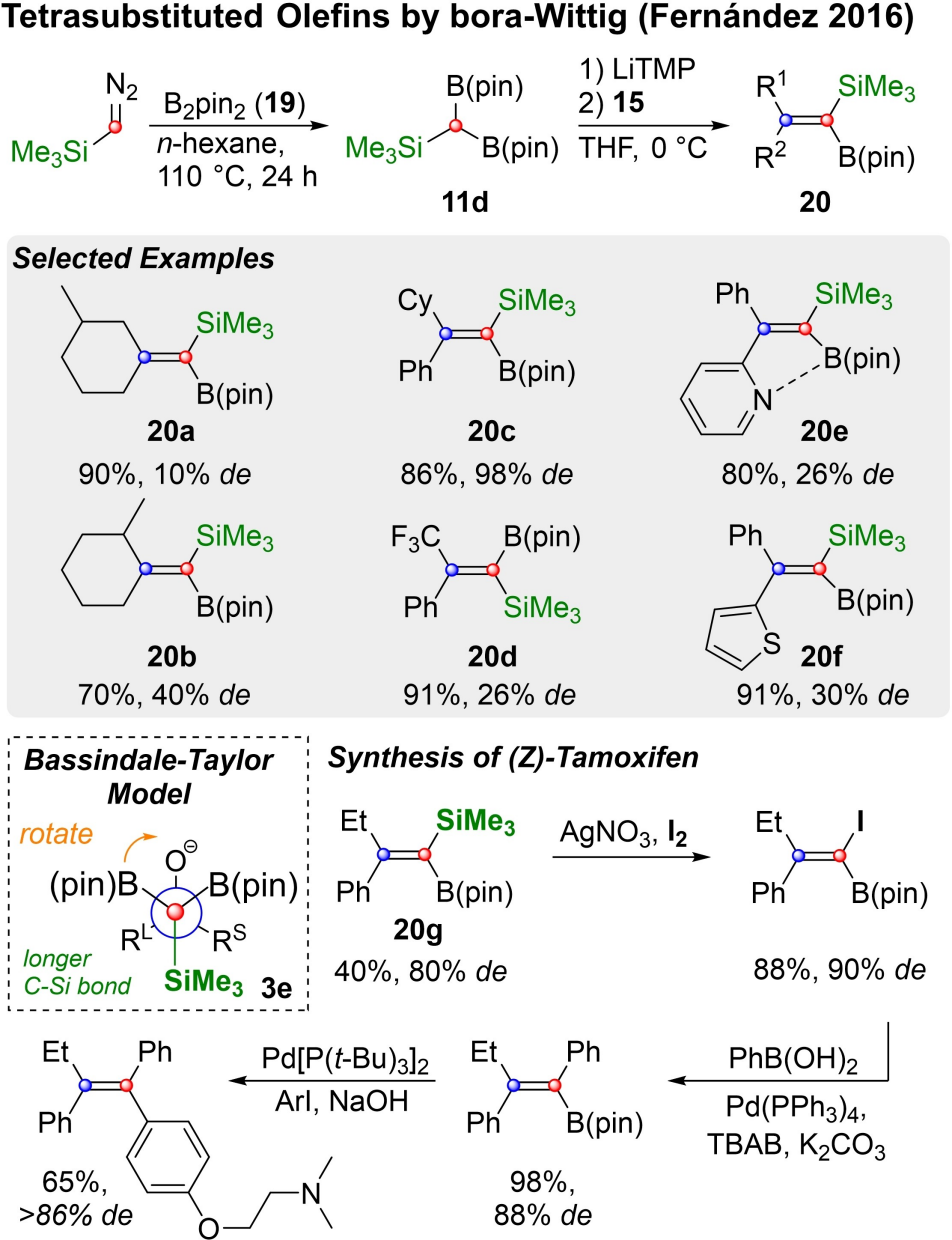
The synthesis of tetrasubstituted alkenes by boron‐Wittig reaction is possible via silyl intermediates.

Thus, Morken established reliable routes to both mono‐ and disubstituted vinyl boronic esters, which are excellent precursors for the synthesis of di‐ and trisubstituted alkenes, as will be discussed in the next chapter. The synthesis of trisubstituted vinylboronic esters through boron‐Wittig reaction and their conversion into tetrasubstituted alkenes was described by Cuenca, Fernández and co‐workers (Scheme 6).^[25]^ Therefore, TMS‐methyldiboronate **11 d**, was prepared by insertion of a TMS‐diazomethane‐derived carbene into B_2_pin_2_ (**19**). The reagent **11 d** was then deprotonated with Li‐TMP and added to different cyclic and non‐cyclic ketones (**15**). This resulted in silanes of type **20**. Good diastereoselectivity was obtained for ketones with a distinguished steric bias, while more remote differences led to diastereomeric mixtures (c.f. **20 a** and **20 b**). In general, the TMS group was preferably located on the side with a bulkier substituent (**20 a**–**d**). Furthermore, a coordinating effect of the 2‐pyridyl substituent in **20 e** was confirmed by ^11^B NMR. In the corresponding 2‐thiophenyl derivative **20 f** no such effect was detected, so a difference in size between the phenyl‐ and the thiophene substituent might be best envoked to explain the moderate selectivity observed for **20 f** (30 % *de*). The Bassindale‐Taylor model predicts the outcome of the reaction of a ketone with sterically different substituents and **11 d** correctly, if one assumes a smaller effective size for the TMS group compared to the boronic esters. Given the greater length of the C−Si bond over the C−B bond^[27]^ this is a reasonable assumption.

Furthermore, Fernández and coworkers showed that silylboronates of type **20** can be funcitionalized directly in one‐pot Suzuki‐Miyaura couplings to substitute the boronate selectively. The silyl group on the other hand can also be selectively converted into the corresponding iodide using I_2_/AgNO_3_. Both reactions can be conducted consecutively as showcased by the authors in their synthesis of *Z*‐Tamoxifen: conversion of **20 g** into the iodide was followed by a Suzuki‐Miyaura coupling, which proceeded in the presence of the pinacol ester.^[26]^ A second Suzuki‐Miyaura coupling led to *Z*‐Tamoxifen under retention of configuration. Fernández and coworkers recently also published an excellent review on boron‐Wittig reactions, which contains numerous examples beyond the scope of this article.^[15]^

### 2.3 Selectivity from Diastereoselective 1,3‐Rearrangement

**Scheme 7:**
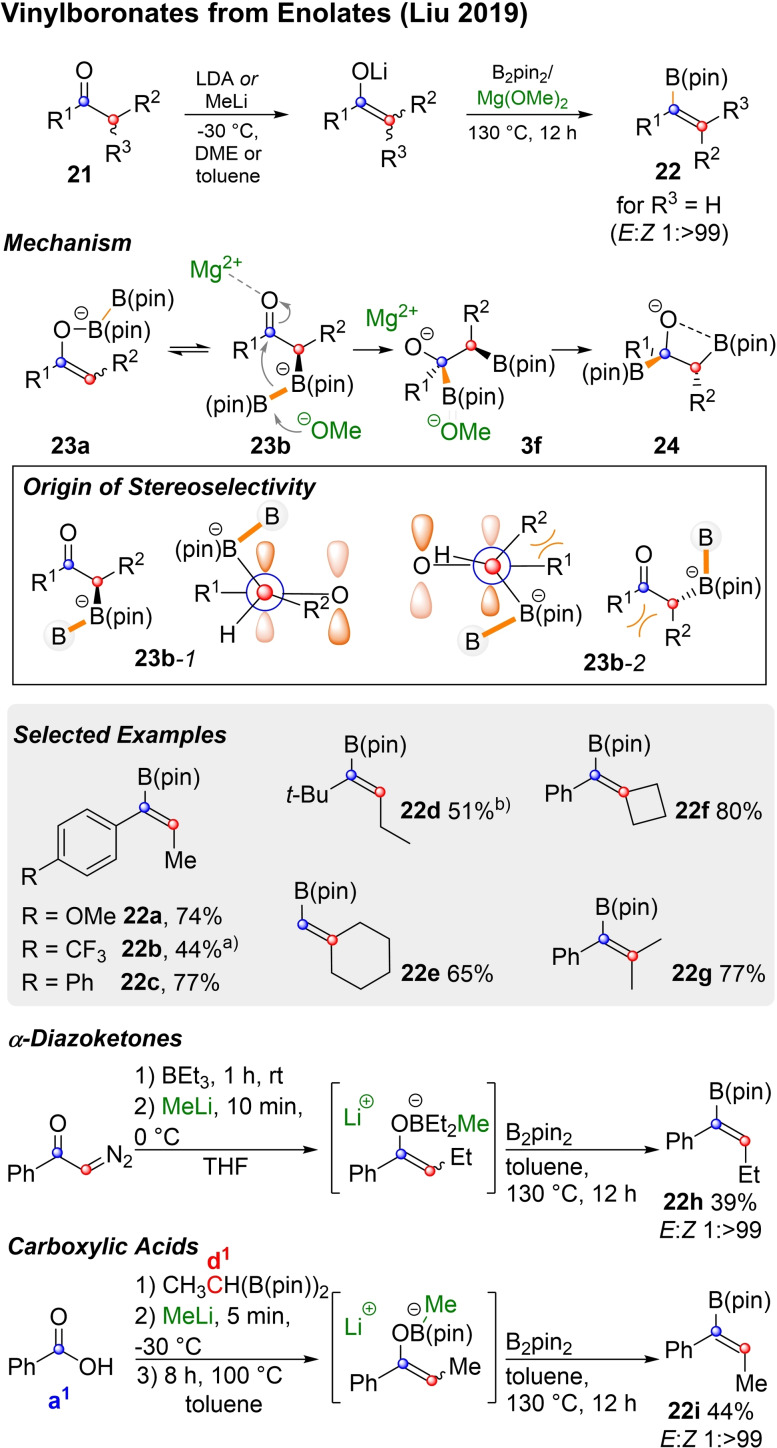
Conversion of enolates into vinylboronic esters and their synthesis from diazoketones and carboxylic acids. a) 150 °C instead of 130 °C. b) Mg(OEt)_2_ instead of Mg(OMe)_2_.

Another approach to trisubstituted vinylboronates, was published by Liu and coworkers^[28]^ and is shown in Scheme 7. Lithiumenolates derived from ketones of type **21**, can be converted into vinyl‐pinacol boronic esters by reaction with bis‐pinacolatoborane and magnesiummethoxide. The enolate initially forms ate‐complexes of type **23** with B_2_pin_2_. The carbon bound ate‐complex **23 b** can undergo a Mg(OMe)_2_ promoted 1,3‐metalate rearrangement. In order for this rearrangement to occure, the migrating B(pin)^−^ group needs to interact with the π*‐orbital of the carbonyl moiety and must thus be transferred from either above (**23 b**‐*1*) or below (**23 b**‐*2*) the C=O‐plane. As **23 b**‐*1* positions R^1^ and R^2^ *anit*‐periplanar from each other, the 1,3‐migration preferably occurs from this conformation and forms **3 f** diastereoselectively. In order to affect *syn*‐elimination **3 f** needs to rotate again, which leads to **24**, from which the alkene is formed, as in a boron‐Wittig reaction.

This way excellent *Z*‐selectivity was achieved for ketones, which are not branched in the α‐position (**22 a**–**d**). Symmetrically branched (i. e. R^2^=R^3^) aldehydes (**22 e**) and ketones (**22 f**–**g**) also delivered good yields and avoided issues of diastereoselectivity. Classically enolate generation does not only suffer from issues of stereo‐ but also regioselectivity. Therefore, Liu's method is compatible with many types of enolate formation, the diastereomeric bias of which is of minor concern, as the overall stereoselectivity originates from the 1,3‐migration. The enolate for **22 e** was generated from the corresponding silylenol ether, while **22 h** was prepared from a diazoketone. For the synthesis of **22 i**, a boron‐enolate was generated form benzoic acid (a^1^) and a d^1^ building block of type **10 c**.^[28]^ Thus, Liu's method does not only provide a highly stereoselective entry into vinyl boronic esters of type **22** (with R^3^=H or R^2^), but also a nice segue into the next paragraph. There, the formation of key elimination precursors of type **3** by 1,2‐metalate rearrangement of boron‐ate‐complexes from three membered heterocycles is discussed.

## 3 Opening Small Heterocycles by 1,2‐Rearrangement

### 3.1 The Zweifel‐Olefination

**Scheme 8:**
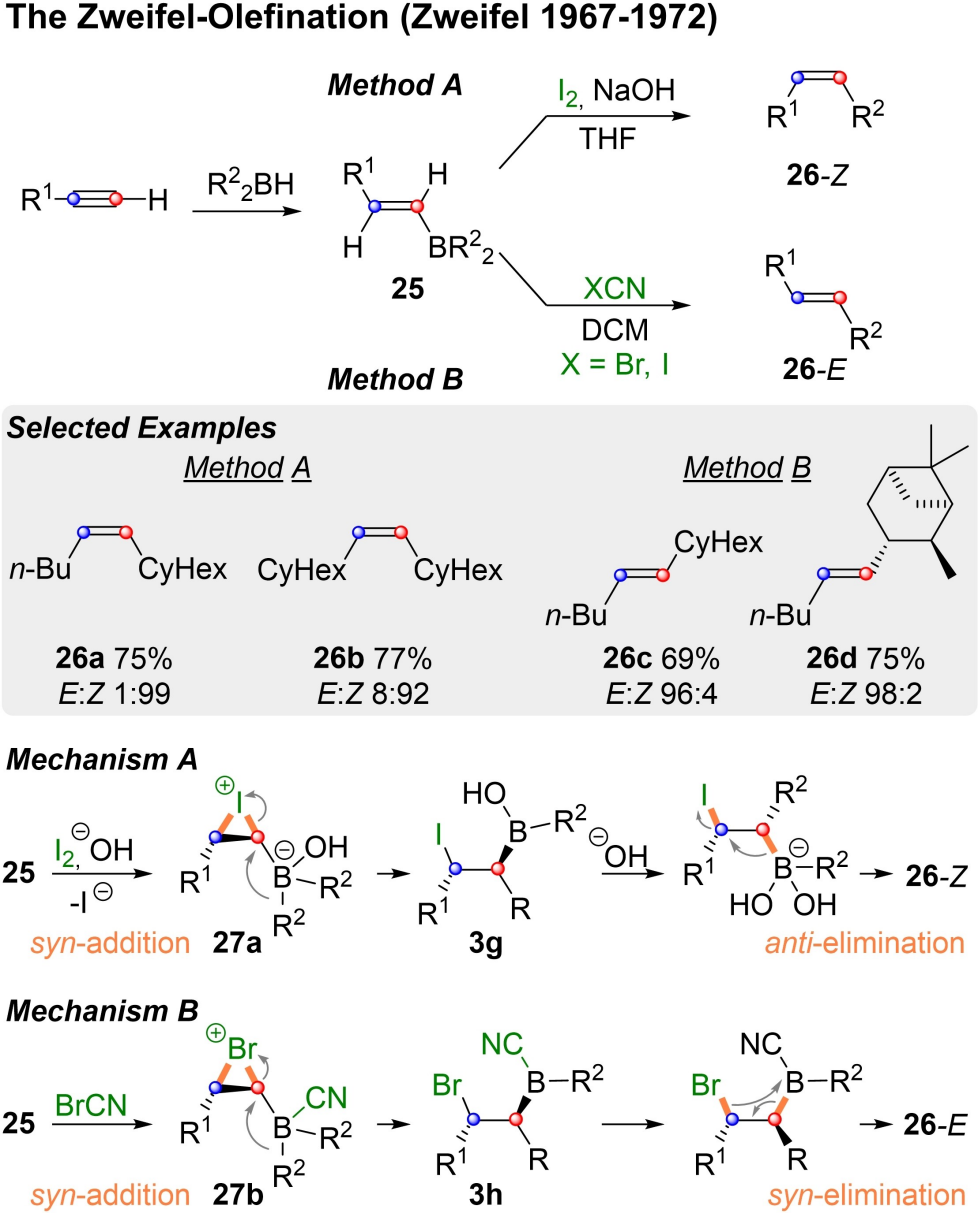
Conversion of vinyl‐boranes into alkenes by Zweifel‐olefination.

In the previous section the conversion of a vinyl‐boronic ester into a highly substituted olefin by Suzuki‐Miyara coupling was shown in Scheme 6. While this famous cross‐coupling is very usefull, it suffers from the usual drawbacks of palladium chemistry (e. g. reduced substrate scope due to competing β‐H‐elimination). Another type of C−C coupling for olefins that proceeds via vinyl‐boron‐ate‐complexes was first reported in 1967 by Zweifel and coworkers.^[30]^ *E*‐Vinylboranes of type **25**, which were derived from alkynes by hydroboration, can be converted into *Z*‐alkenes (**26 a/b**), by addition of iodide and sodium hydroxide in THF (Scheme 8, Method A). When the reaction is conducted in DCM with a halogen cyanide *E*‐alkenes like **26 c** and **26 d** were obtained (Method B).

In both cases the reaction proceeds via a cyclic halonium ion of type **27**, in which the borane has formed an ate‐complex. Subsequent 1,2‐metalate rearrangement leads to elimination precursors **3 g/h**. The key difference between the two methods lies in the type and amount of base employed. In mechanism A **3 g** can react with excess hydroxide to a new ate‐complex, which undergoes *anti*‐elimination to **26**‐*Z*. In mechanism B on the other hand no strong base is present and thus, the cyanide anion remains bound to the boron atom. The resulting electrondeficient borane **3 h** then undergoes *syn*‐elimination. The pivotal ate‐complexes of type **27** do not necessarily have to be formed from vinyl‐boron derivatives.

**Scheme 9:**
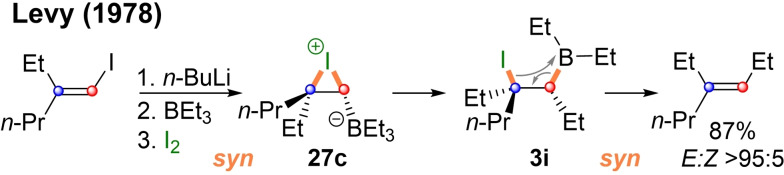
Conversion of vinyl‐metalates into alkenes by Zweifel‐olefination.

As shown in Scheme 9 the vinyl‐group can also be introduced as a lithium species, which is reacted with a saturated borane and then converted into the key rearrangement precursor **27 c** by addition of iodine.^[31]^

**Scheme 10:**
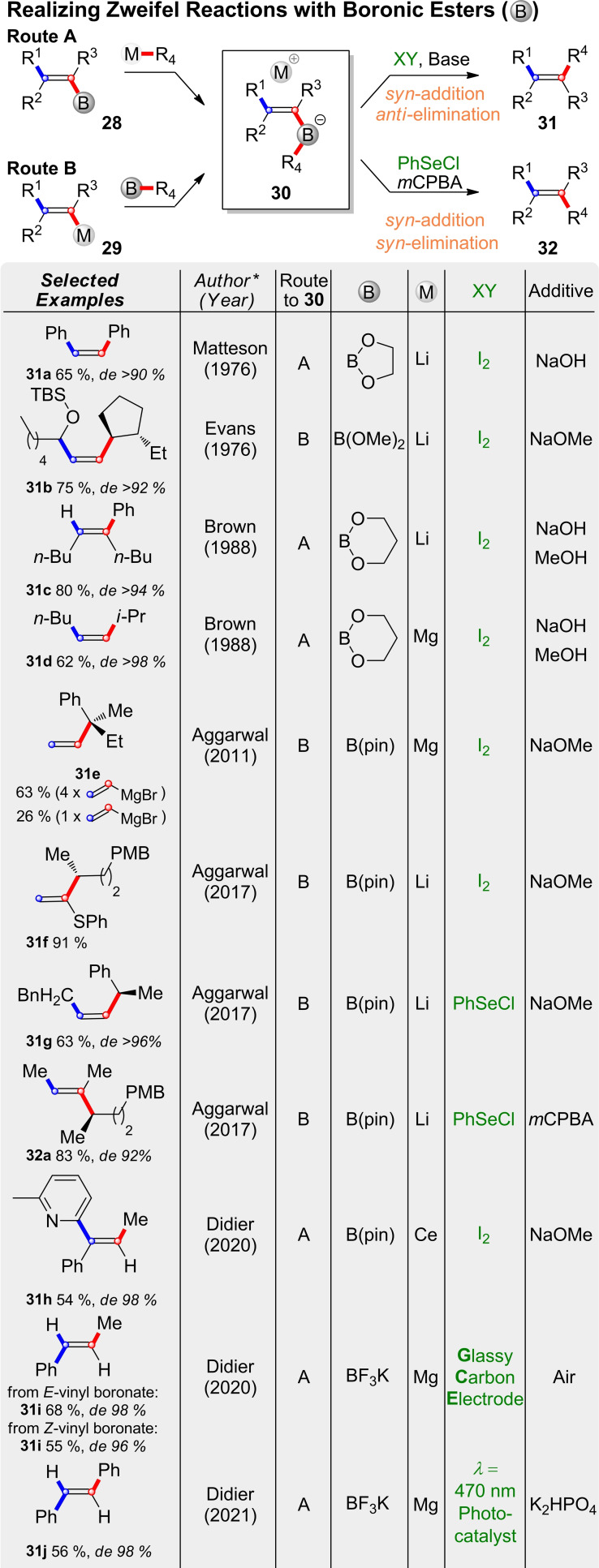
Conversion of boronic esters into alkenes by Zweifel‐olefination.

Thus, pivotal features of the Zweifel‐olefination have been demonstrated quite early, but an inherent problem of boranes as substrates lies in the transfer of only one of the borane's alkyl groups. To some degree this limitation can be mitigated by the use of borinic esters.^[32]^ However, modern Zweifel‐reactions mostly employ the more stable boronic esters as shown in Scheme 10, in which the key strategies of the last 45 years are summarized. Vinylboronic esters (**28**) can be reacted with different alkyl or aryl metal species to vinyl‐ate‐complexes of type **30** (Route A). However, the same complexes can also be prepared by addition of a vinyl metal species (**29**) to an alkyl boronic ester (Route B). In both cases subsequent *syn*‐addition of an electrophile followed by base induced *anti*‐elimination leads to olefins of type **31** under overall inversion of configuration. Both approaches have been realized with alkyl lithium reagents in 1976 by Matteson^[22]^ (**31 a**) and Evans^[33]^ (**31 b**). In 1988 Brown and coworkers reported the synthesis of tertiary alkenes (**31 c**) and the use of alkyl Grignards (**31 d**).^[32]^

**Scheme 11:**
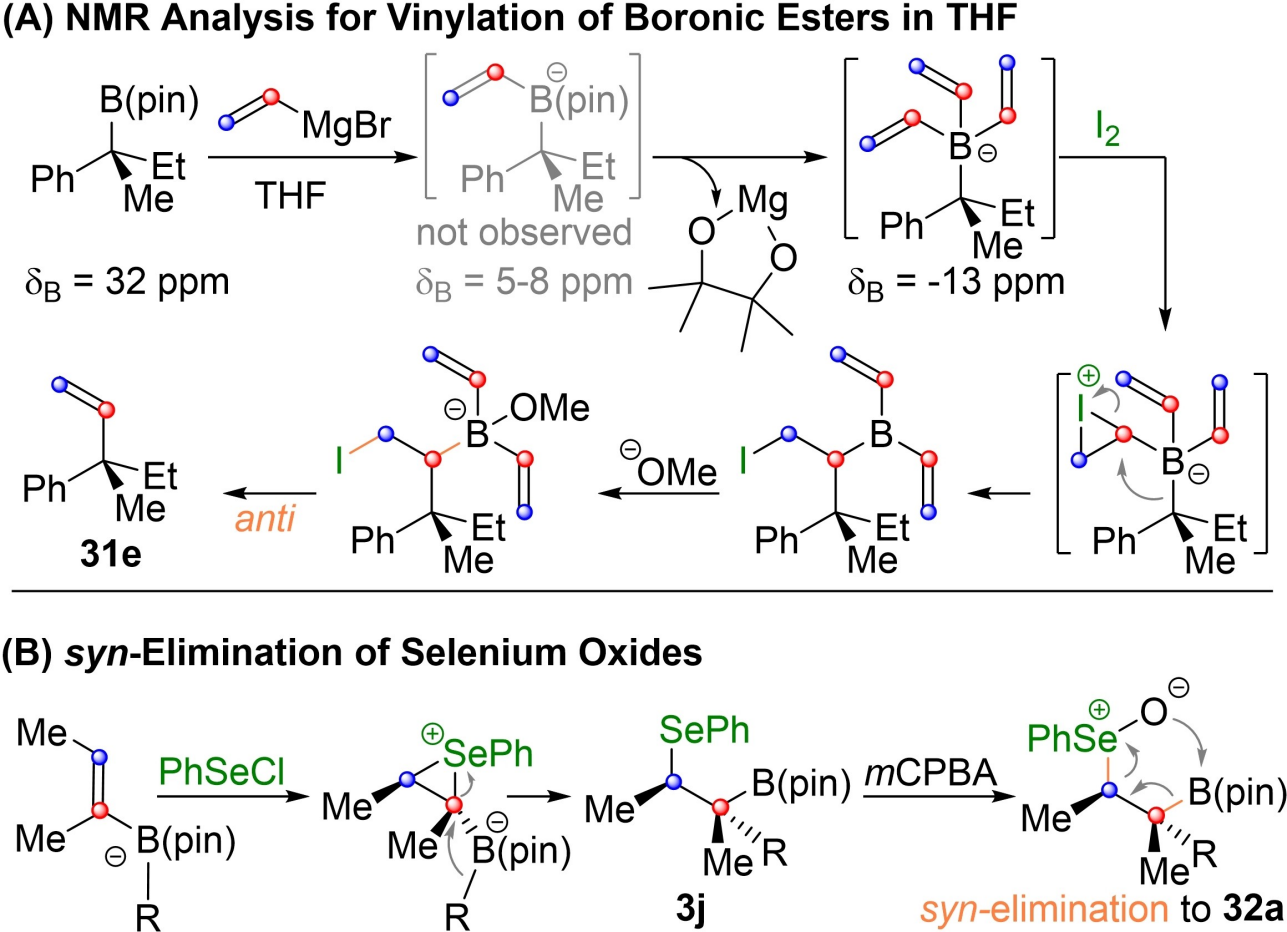
Mechanistic details related to Scheme 10.

However, the use of Grignard reagents can be tricky for the introduction of vinyl groups. In 2011 Aggarwal and coworkers reported optimized conditions for Zweifel‐olefinations with vinyl metal species (**31 e**).^[34b]^ As magnesium forms a stable chelate complex with pinnacol, the reaction with vinyl magnesium bromide can lead to a trivinyl‐borate species (Scheme 11A).^[34]^ Although this intermediate can also react to the desired olefination product, it requires three equivalents of vinyl magnesium bromide to do so. In practical terms this results in low yields when only one equivalent of the reagent is used. In the case of primary and secondary substrates this can be remedied by the addition of DMSO, while tertiary substrates require the use of an excess of vinyl Grignard (**31 e**). Sterically extremely hindered substrates are best vinylated with the harder vinyl lithium, which can be conveniently prepared from the corresponding stannane. In 2017 the Aggarwal group also reported the use of heteroatom substituted vinyl lithium species (**31 f**, Scheme 10)^[34]^ and a variation of the Zweifel‐olefination using phenylselenyl chloride as an electrophile.^[35]^ The 1,2‐metalate rearrangement produces selenylethers of type **3** (X=SePh), which can undergo base induced *anti*‐elimination in the usual manner. Like in standard Zweifel‐conditions (I_2_, NaOR) this leads to overall inversion (**31 g**, Scheme 10). However, oxidation of the selenylether **3 j** (Scheme 11B) to the selenoxide with *m*CPBA fostered *syn*‐elimination. Thus, the overall reaction proceeded under retention (**32 a**). This procedure provides a significant extension for Zweifel‐olefinations of boronic esters, as they are more electron rich and thus do not undergo *syn*‐elimination with halocyanides.

Thus, Aggarwal's 2017 protocol completes the set of Zweifel‐olefinations for boronic esters (top part of Scheme 10). Now both, vinyl‐boronic esters and vinyl metal species, can be converted into substituted olefins under inversion or retention with high stereochemical fidelity. A more detailed discussion of the Zweifel‐reaction can be found in an excellent review published by Armstrong and Aggarwal in 2017.^[35]^

Since then the Didier group has used Zweifel‐olefinations for a variety of four membered (hetero)‐cycles^[36]^ and significantly extended the method by employing aryl‐ as well as vinyl cerium species (Route A and B (Scheme 10), respectively).^[37]^ These milder reagents allowed for an impressive substrate scope and tolerance of functional groups including heterocycles (**31 h**, Scheme 10), esters, amides and nitriles. In the same year the group also reported an electrochemical variant of the Zweifel‐olefination. Here the addition of an electrophile is replaced by oxidation at a glassy carbon electrode (GCE).^[38]^ The resulting radical cation can also undergo 1,2‐metalate rearrangement, after which oxidation by air delivers alkenes such as **31 i**. Due to the cation radical intermediate the reaction loses its stereofidelity, but preferably produces the thermodynamically more stable alkene. One year later the group published a photocatalytic conversion of vinyl triarylborate complexes to alkenes in the presence of K_2_HPO_4_ and irradiation with blue light (**31 j**).^[39]^ Under related conditions overoxidation of the products to stilbeneoxides was observed. Thus, it is likely that the reaction proceeds via epoxide inertmediates that undergoe Zweifel‐type rearrangements and subsequent elimination.

**Scheme 12:**
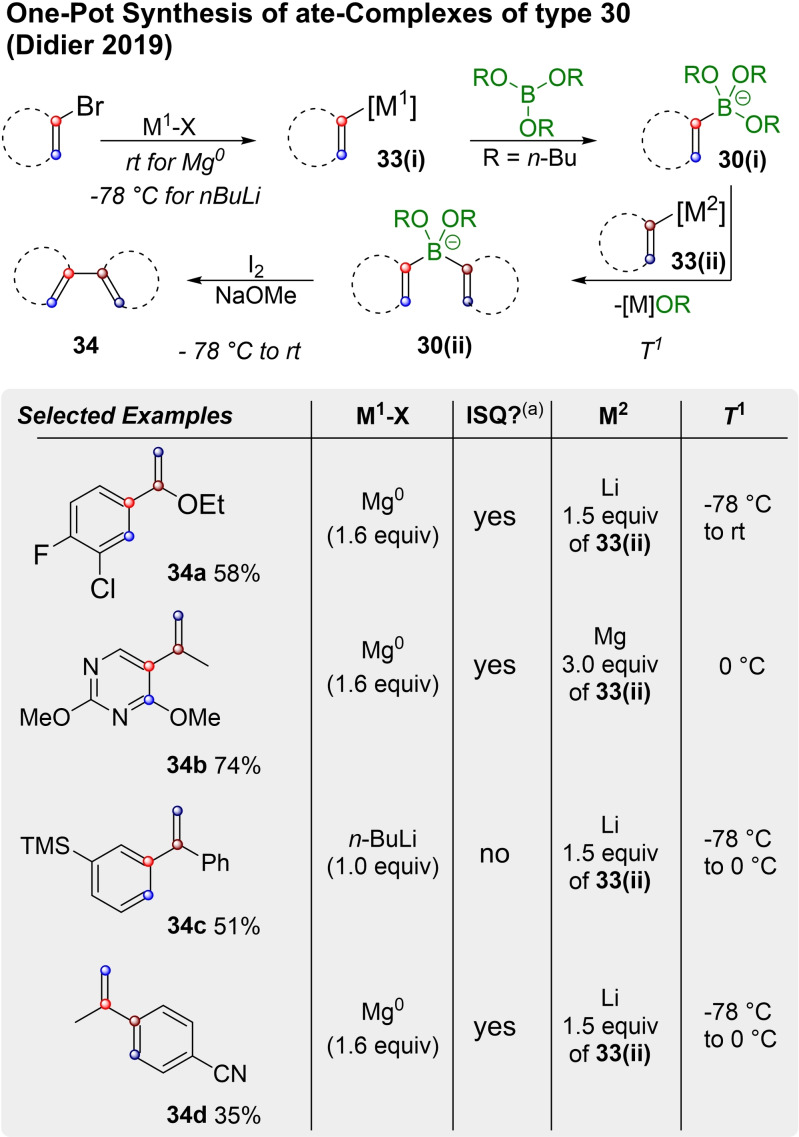
One‐pot synthesis of boronates for Zweifel‐olefination. (a) ISQ=in situ quench (i. e. **33(i)** is generated in the presence of the borate).

Another convenient extension of the method was reported by the Didier Group in 2019,^[40]^ in which preparation and isolation of the boronic ester previous to the Zweifel‐olefination is omitted. As shown in Scheme 12 aryl‐, hetereoaryl‐ or vinyl bromides are converted into vinyl lithium or vinyl magnesium species of type **33(i)**, which are reacted with tri‐*n*‐butyl borate to **30(i)**. Classically, **30(i)** would be converted into a boronic ester, which is isolated and reacted with a second organo metal species in a subsequent Zweifel‐reaction. Didier, however, optimized a variety of condition sets that allow for in situ reaction with another organo metal species of type **33(ii)**, thus forming ate‐complexes like **30(ii)**, which can complete the Zweifel‐olefination (to **34**) directly.

### 3.2 Epoxide Olefination

**Scheme 13:**
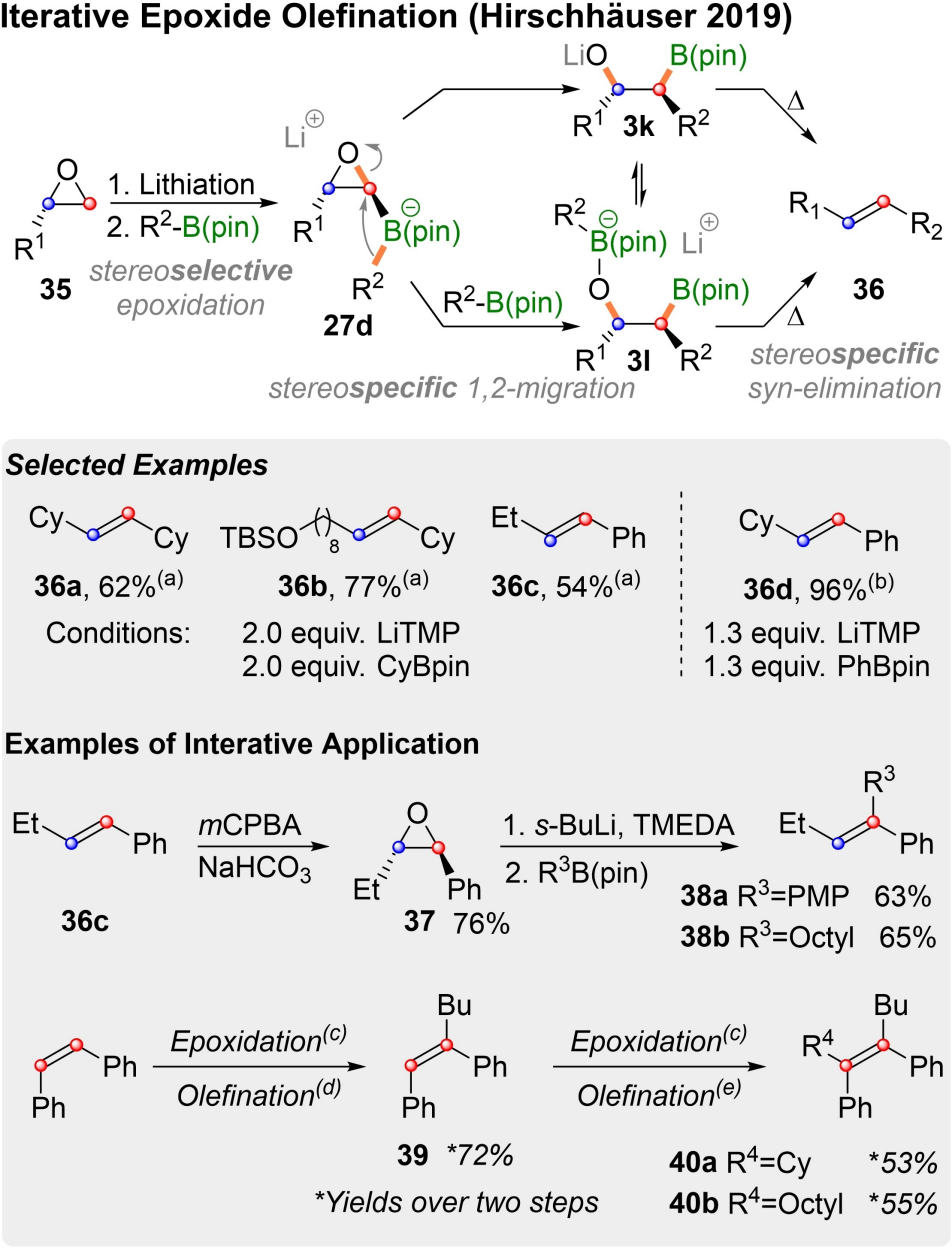
Epoxide olefination and iterative application. (a) LiTMP (THF, 0 °C, 2 h), to rt, elimination (60 °C, 2 h). (b) LiTMP (THF, 0 °C, overnight), to rt, elimination (60 °C, 2 h). (c) *m*CPBA/NaHCO_3_ (DCM, overnight). (d) *n*‐BuLi/TMEDA (THF, −98 °C, 0.5 h), BuB(pin) (60 °C, overnight). (e) *t*‐BuLi/TMEDA (Et_2_O, −78 °C, 0.25 h), R^4^B(pin) (38 °C, overnight).

For Zweifel‐olefinations rearrangement precursors of type **27** are generated by addition of electrophiles to vinyl‐ate‐complexes **30**. An alternative route was realized by us in 2019 through the reaction of lithiated epoxides with boronic esters (Scheme 13).^[41]^ Shimizu,^[42]^ Blakemore^[43]^ and Aggarwal^[44]^ had described the insertion of lithiated epoxides into boronic esters, which proceeds through ate‐complexes **27 d** and leads to alkoxides **3 k**. To foster olefin formation, we initially attempted to mimic Pelter's approach and react **3 k** with different acid chlorides. This led to unsatisfactory mixtures of *E*‐ and *Z*‐alkenes, as these esters could undergo both *syn*‐ and *anti*‐elimination. However, thermal *syn*‐elimination of the unmodified alkoxides led directly to alkenes (**36**). As reported by Hodgson et al.^[44c]^ lithiation of epoxides of type **35** proceeds with extremely high stereoselectivity. As all subsequent processes in the sequence are stereospecific, this initial stereoselectivity is translated into virtually isomerically pure olefins (**36 a**–**c**). A curious aspect of the reaction is the need for two equivalents of saturated boronic esters to obtain satisfactory yields (e. g. **36 a**).

The excess boronic ester could promote the metalate rearrangement, by acting as a Lewis acid. However, so far, our attempts to substitute it by an external Lewis acid have failed. Excess boronate could also form an ate‐complex of type **3 l** with the alkoxide **3 k**. Alter elimination of **3 l** to **36** the boronic ester would be liberated, but if this occurs after the lithiated epoxide has already decomposed, low yields are to be expected. Later it was found,^[45]^ that only 1.3 equiv. of aromatic boronic esters (e. g. R^2^=Ph) are necessary for the synthesis of styrene derivatives such as **36 d**. This fits with the latter mechanistic interpretation, as elimination to the conjugated olefin can be expected to occure much more swiftly to the conjugated alkene. An important feature of this olefination lies in it's iterative applicability. By subjecting olefins such as **36 c** to stereospecific Prileschajew epoxidation^[46]^ corresponding higher substituted epoxides can be generated. Lithiation of disubstituted epoxides can generate issues of regioselectivity, but styrene oxides such as **37** can be selectively lithiated in the benzylic position and reacted to trisubstituted alkenes of type **38**. Tetrasubstituted alkenes were synthesized in an iterative manner from *cis*‐stilbene. Epoxidation and olefination with BuB(pin) delivered the trisubstituted olefin **39** and upon iteration tetrasubstituted alkenes of type **40**. However, it should be mentioned that an increasing number of substituents can impair epoxide lithiation and thus olefination. This problem was encountered for example in attempts to replicate the sequence to olefins of type **40** starting from *trans*‐stilbene. There, all our attempts for a second iteration failed completely.

**Scheme 14:**
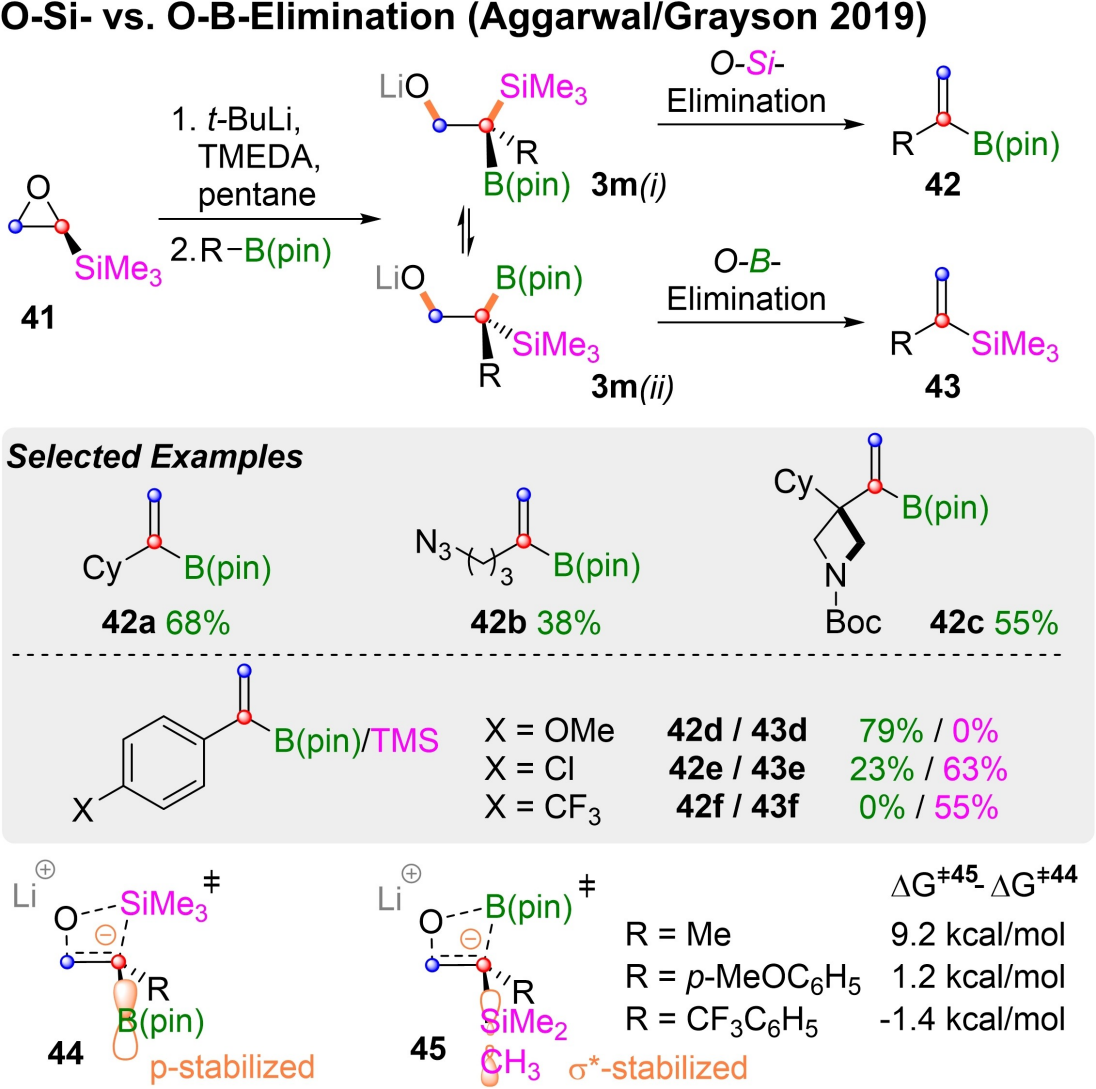
Synthesis of vinyl boronic esters/vinyl silyl compounds.

In the same year Aggarwal, Grayson and coworkers^[47]^ studied the reaction of lithiated TMS‐oxirane (**41**) with boronic esters (Scheme 14). Regioselective lithiation of **41** occures in α‐position to the silyl group. Insertion of the resulting carbenoid into boronic esters generates alkoxides of type **3 m**, in analogy to Scheme 13. These alkoxides can undergo either a Peterson‐type O−Si‐elimination to a vinylboronic ester of type **42** or Boron‐Wittig‐type O−B‐elimination to vinyl silanes of type **43**.

The desired vinyl‐boranes of type **42** were obtained with sterically hindered secondary (**42 a**) and tertiary boronic esters (**42 c**) in good yields. Primary boronates with functional groups that are sensitive to base and/or nucleophiles generated only moderate yields (**42 b**). Electron rich aryl boronic esters also preferably formed vinyl‐boronates like **42 d**. However, more electrondeficient aryl boronic esters showed a greater tendency for O−B‐elimination to vinyl silanes (c.f. **43 e** and **43 f**). This raises the question, which factors favor O−Si over O−B‐elimination or *vice versa*. Quantummechanical calculations showed that vinyl silanes of type **42** are thermodynamically more stable than the corresponding vinyl boronic esters **43**, if R is sp^3^‐ or sp^2^‐hybridized. Calculations of the relevant transition states revealed that O−Si‐elimination is favored by 9.2 kcal/mol for R=Me. This concurs with chemical intuition, as the empty p‐orbital of the boron‐atom is better able to stabilize the developing negative charge in **44**, than the highlighted σ*‐orbital of the TMS group in **45**. However, as more electron deficient substituents (R) also provide stabilization for the newly forming carbanion, the higher lewis acidity of the boron atom favors O−B‐elimination via transition state **45**.

Looking back at the methods discussed so far numerous synergies immediately become apparent that could be exploited for the stereoselective synthesis of highly substituted olefins. Boron‐Wittig reactions have been developed to a point, where they give convenient access to highly substituted vinyl‐boronic esters. The selectivity of these reactions can be predicted by the Bassindale‐Taylor model. Such vinyl‐boronic esters could be subjected to Zweifel‐olefinations, which allow for substituting the boronate with an alkyl metal species. More importantly the overall configuration of the final product can be chosen freely at this stage by promoting either *syn*‐ or *anti*‐elimination. Finally, an epoxidation/olefination sequence could be employed for further substitution. However, at some point of every process, that employs achiral startingmaterials to synthesize olefins, a stereo‐ or regioselective step must occure, which is dependent on significantly different substituents. Thus, the seemingly simple example **1 a** (ethyl‐propyl‐butyl‐pentyl‐ethylene) shown in Scheme 1 would still be difficult to make this way. In the next section we will thus look at a somewhat different strategy, which relies on the synthesis of enantiomerically pure precursors of type **3** and their stereospecific elimination.

## 4 Boron compounds as a^1^‐synthons

**Scheme 15:**
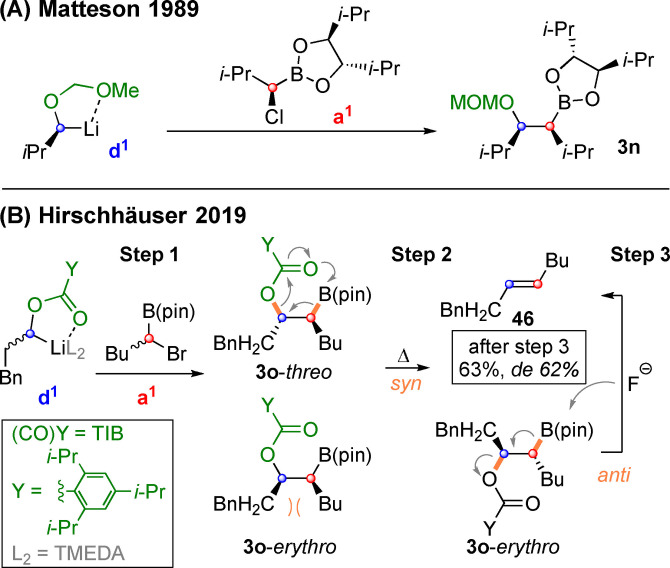
Matteson‐substitution with chiral carbenoids and subsequent boron‐Wittig elimination.

Lately, an umpolungs strategy has emerged, that employes chiral carbanions that are combined with boronic esters carrying a leaving group in the α‐position. As shown in Scheme 15A substitution of a chiral a^1^ reagent, i. e. an α‐chloro boronate and a chiral d^1^ reagent, i. e. a MOM stabilized carbanion, had already been reported by Matteson in 1989.^[48]^

We currently attempt to extend this chemistry to alkyl lithium species, derived from carbamates and sterically hindered esters. Such chiral carbanions can be generated much more easily in scalemic form by Hoppe‐lithiation with spartein or other chiral diamine ligands.^[49]^ However, the resulting coupling products of type **3 o** are quite prone to elimination (Scheme 15B). Interestingly, even racemic combinations of carbanions and α‐halo boronates preferably deliver *E*‐Alkenes such as **46** upon elimination.^[41]^ The reaction proceeds via a mixture of diastereomeres of type **3 o** (and their enantiomers). The *syn*‐periplanar transition state for the *syn*‐elimination of **3 o**‐*threo* is sterically less hindered than the one for **3 o**‐*erythro*. Thus, the *E*‐alkene **46** is preferably formed. Through addition of fluoride *anti*‐elimination of remaining **3 o**‐*erythro* could be initiated and thus **46** was formed in 63 % yield with 62 % *de*. Our experiments in this particular area were rather of mechanistic than of preparative interest. Especially as Blakemore and coworkers^[50]^ had already realized a highly predictable synthesis of teriary alkenes by employing enantiomerically pure starting materials.

**Scheme 16:**
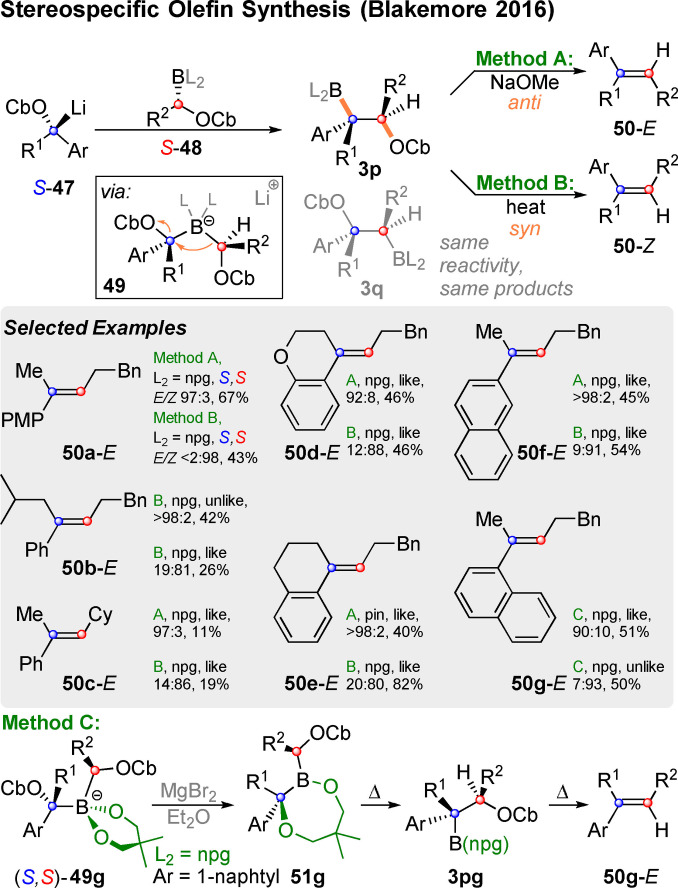
Stereospecific synthesis of styrene derivatives via *syn*‐ or *anti*‐elimination.

Their reaction of lithiated benzylic carbamates **47** with a neopentylglycol (npg) or pinacol boronic ester of type **48** is shown in Scheme 16 and proceeds in three stereospecific steps: The formation of a boron‐ate‐complex **49** (Step 1) is followed by a 1,2‐metalate rearrangement under elimination of one of the two carbamates (**3 p**, Step 2). In this anionotropic rearrangement both carbamates could theoretically serve as leaving groups. In this case, the non‐benzylic anion (red) migrates preferably, so that **3 p** is formed instead of **3 q**. Most elimination precursors of type **3 p** are sufficiently stable, so that in step 3 the operator can choose whether to conduct an *anti*‐elimination by adding NaOMe (yielding **50**‐*E*), or a *syn*‐elimination by heating without additional base (yielding **50**‐*Z*).

Thus, there are two pivotal points in the sequence, at which the overall configuration of the alkene **50** can be choosen. Firstly, by selecting an appropriate combination of enantiomers of **47** and **48**, a “like” combination of *S*‐**47** and *S*‐**48** or *R*‐**47**, *R*‐**48** produces **3 p** or its enantiomer *ent*‐**3 p**, respectively. Both enantiomers can form the same alkenes of type **50** under the same conditions. An “unlike” pairing (*S*‐**47** and *R*‐**48** or *R*‐**47** and *S*‐**48**) leads to one of two epimers of **3 p**, which have complementary reactivity. Now **50**‐*Z* would form upon *anti*‐elimination with NaOMe and **50**‐*E* would form upon thermal *syn*‐elimination. Both enantiomers of carbamates **48** are readily available via Hoppe‐lithiation with the appropriate diamine. This is already sufficient for selecting the desired olefin configuration.^[49]^ Secondary carbamates such as *S*‐**47** can be prepared by a variety of methods.^[51]^ As examples **50 a**–**50 f** demonstrate, a wide range of styrene derivatives can be prepared in this manner. Given the sterically hindered nature of ate‐complexes of type **49** the sterically less hindered neopentylglycol esters often deliver better results than their pinacol analogues. In the case of the sterically highly crowded 1‐naphtyl derivative **49 g** competing O‐migration to borinate **51 g** was observed. By adding MgBr_2_, this transformation was achieved with high stereochemical fidelity and heating of **51 g** finally lead to the desired C‐migration (**3 pg**) and subsequent *syn*‐elimination to **50 g**‐*E*. While this side reaction does not allow for choosing the desired configuration at the elimination stage anymore, both **50 g**‐*E* and **50 g**‐*Z* were obtained by appropriate like or unlike combination of starting materials. In the meantime, this method has been applied by Blakemore and co‐workers to the synthesis of a p‐glycoprotein inhibitor and its isomere.^[53]^ The overall concept of coupling carbenoids in order to make alkenes was discussed by Hoffmann and Blakemore in an interesting review article.^[53]^

## 5 Conclusions and Outlook

**Scheme 17:**
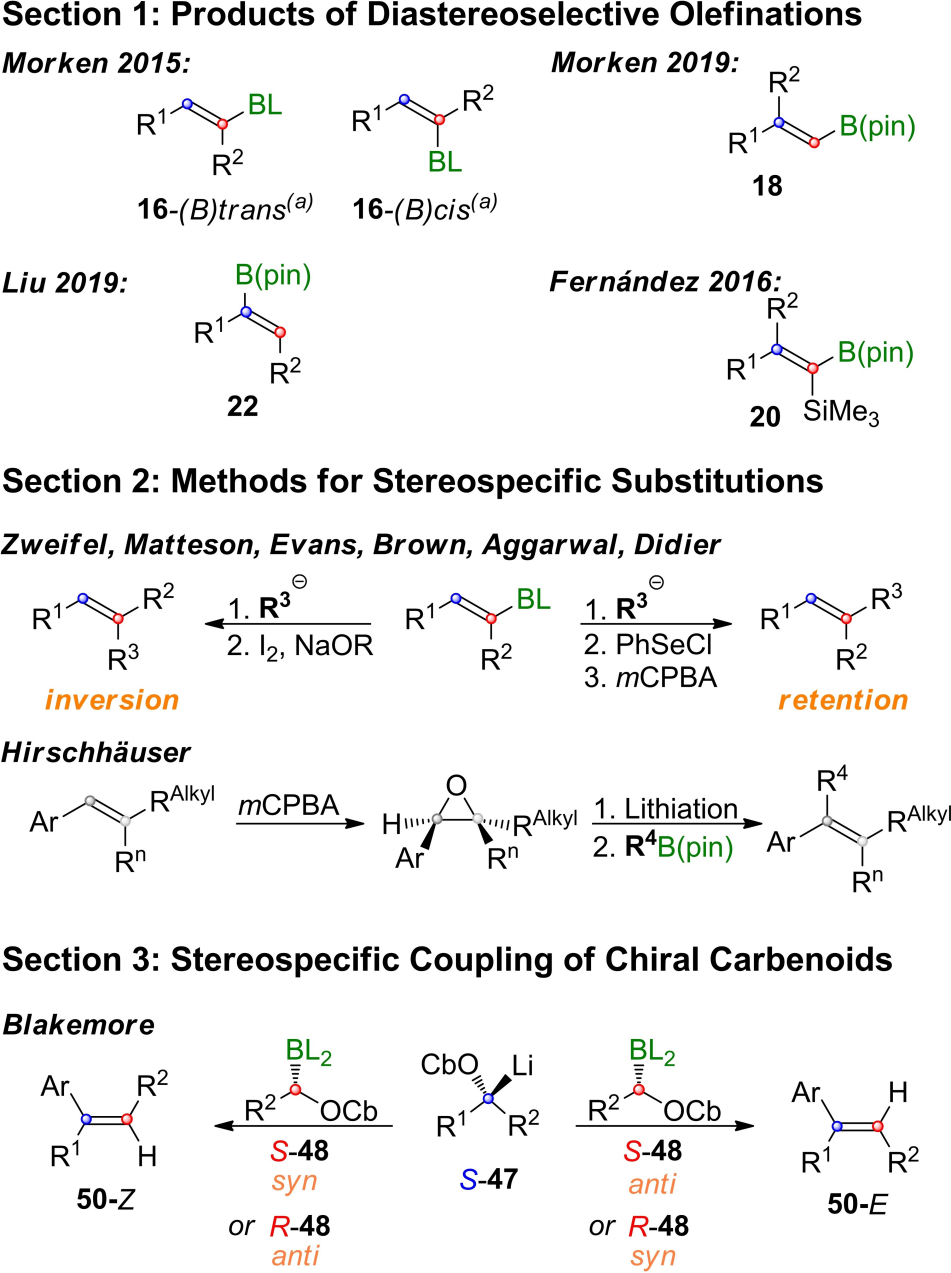
Overview of motives available by the methods discussed in this review.

Olefinations based on the stereoselective generation of elimination precursors of type **3** have seen tremendous development in the last decade. In order to conclude this review, we have comprised the most recent methods discussed in Scheme 17, from which some interesting opportunities for combinations become apparent. As discussed in section 1, a wide variety of disubstituted vinylboronic esters have become available by the diastereoselective methods developed by Morken, Liu and Fernandez (section 1).

In section 2 we saw that such vinylboronic esters are excellent substrates for Zweifel‐olefinations, which now allows for choosing, whether the boronate is substituted under retention or under inversion. Therefore, it does not matter, which diastereomere (e. g. of type **16**) is preferably formed by diastereoselective olefination, as long as it is formed with a decent *de*. Finally, the trisubstituted olefins prepared this way could be converted into epoxides, which after lithiation can be converted into tetrasubstituted olefins. Unfortunately, lithiation of such highly substituted epoxides has a somewhat limited substrate scope.

In section 3 we encountered boronic esters as means to fuse chiral carbenoids into highly substituted olefins. Such carbenoids can be prepared from primary or secondary alcohols, which are protected as carbamates or triisopropylbenzoates.^[54]^ While it might seem excessive to synthesize two chiral compounds in order to prepare an achiral olefin, this approach bears the potential to serve as an unified strategy for synthesizing even the most challenging olefins (such as **1 a**).

**Scheme 18:**
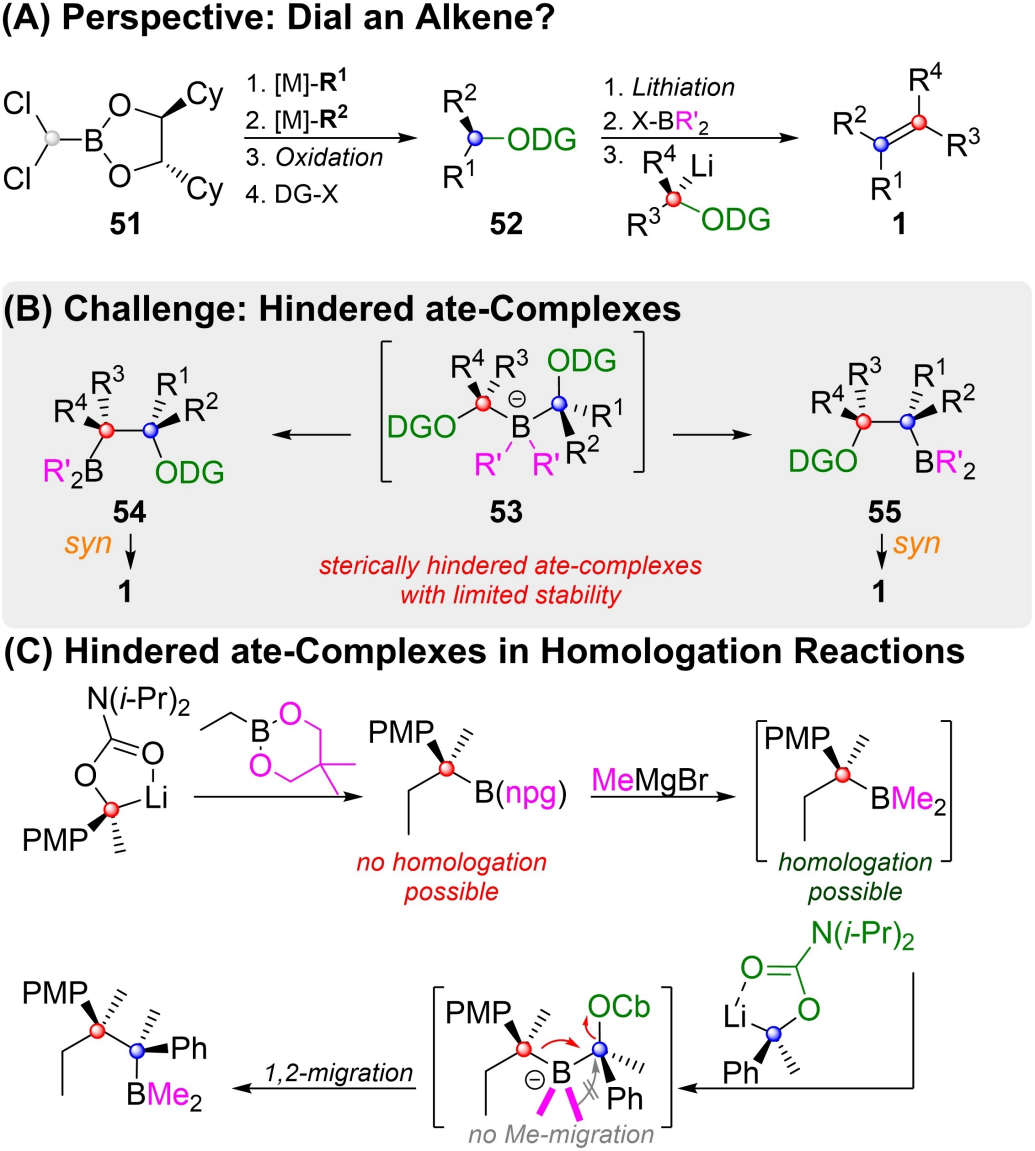
Outlook and challenges.

One potential route is depicted in the outlook‐Scheme 18A. Secondary alcohols are easily prepared by Matteson‐homologation of boronic esters,[^13^, ^55^] or by subsequent substitution and oxidation of **51**.[^56^, ^57^] Protection with a suitable directing group (DG) would deliver carbenoid precursors of type **52**. If two carbenoids derived from **52** could be coupled, it might be possible to arrange at will four organometallic reagents around a stereochemically pure, tetrasubstituted olefin.

However, in order to achieve this lofty goal, some challenges still have to be overcome (Scheme 18B). Blakemore used benzylic, disubstituted carbenoids derived from carbamates (**47**), which are comparably easy to generate. However, Aggarwal has shown that non‐benzylic disubstituted carbenoids are available from triisopropylbenzoates of type **52** (R^1^/R^2^=Alkyl, DG=TIB).^[54]^ Another problem for the application of Blakemore's method to tetrasubstituted alkenes lies in the high steric hindrance ate‐complexes of type **53** would suffer. However, similarly crowded ate‐complexes are formed as intermediates, when two adjacent quarternary stereocenters are generated by a boronate homologation. Aggarwal and co‐workers were able to overcome the steric hindrance in this type of reaction by converting the boronic ester into a dimethylborane (Scheme 18C).^[58]^ Competing 1,2‐migration of the methylgroups were of some concern, however, a clear preference for the tertiary (red) carbon atom was observed. In the proposed ate‐complex **53** it does not matter which of the former carbenoids (red vs. blue) undergoes 1,2‐migration, as long as no competing migration of R’ takes place. Both carbenoid rearrangement products (**54** and **55**) should deliver the same alkene upon *syn*‐elimination. So, could conversion into a borane work for Blakemore's olefination as well?

All in all, recent advances in boron‐based olefinations have focused heavily on controlling the elimination of boronic esters of type **3**. At the same time strong advances are being made in the preparation of such motives by boronate homologation chemistry.^[13]^ Currently we do not yet live in a world, in which every tetrasubstituted olefin can be prepared in an isomerically pure fashion, for example by assembeling four Grignard reagents around two carbenoids. However, it can at least be said that such a world is not inconcievable anymore.

## Conflict of interest

The authors declare no conflict of interest.

6

## Biographical Information

*Kevin Bojaryn studied chemistry at the university of Duisburg‐Essen and received his Master degree in 2018, investigating the syntheses of highly substituted alkenes from boronates. During his PhD thesis in the Hirschhäuser Group, he works on new synthetic routes for the asymmetric synthesis of orthogonally protected vicinal diols*.

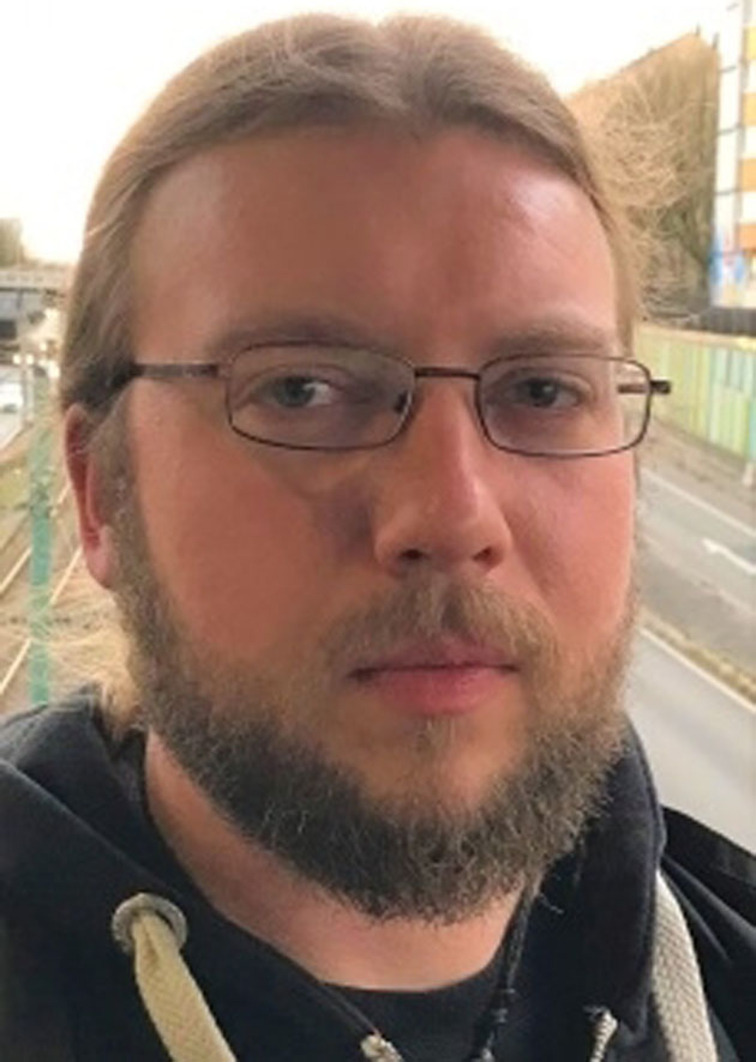

## Biographical Information

*Christoph Hirschhäuser studied Chemistry in Cologne, where he received his PhD for work in the Schmalz Group on iron‐containing nucleoside analogues (2008). Afterwards he went to Bristol for a postdoctorate, in the Gallagher Group where he worked on natural product synthesis and later for AstraZeneca. In 2012 he came to Essen where he became a permanent employee in 2014. His independent research group focusses on new applications for boronic esters in stereoselective synthesis*.

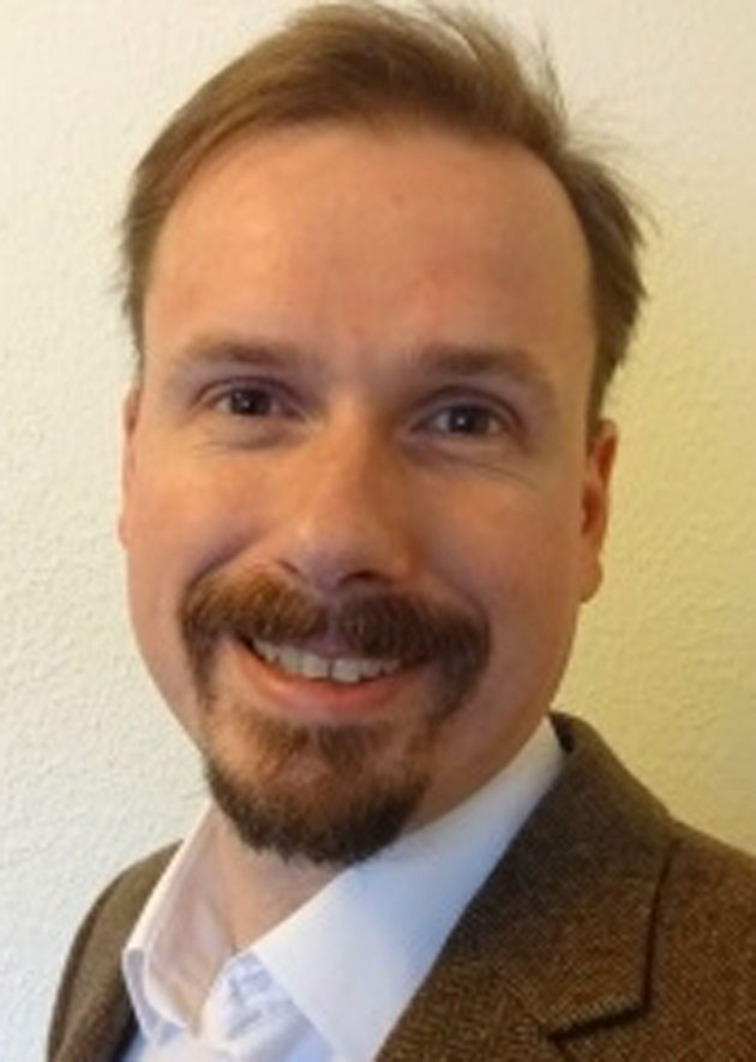

## Data Availability

Data sharing is not applicable to this article as no new data were created or analyzed in this study.
